# Human Immunodeficiency Virus type-1 reverse transcriptase exists as post-translationally modified forms in virions and cells

**DOI:** 10.1186/1742-4690-5-115

**Published:** 2008-12-18

**Authors:** Adam J Davis, Jillian M Carr, Christopher J Bagley, Jason Powell, David Warrilow, David Harrich, Christopher J Burrell, Peng Li

**Affiliations:** 1 Infectious Diseases Laboratories, SA Pathology, Adelaide 5000, Australia; 2 School of Molecular and Biomedical Science, University of Adelaide, Adelaide 5005, Australia; 3 Adelaide Proteomics Centre, University of Adelaide, Adelaide 5005, Australia; 4 Division of Human Immunology, SA Pathology, Adelaide 5000, Australia; 5 Division of Infectious Disease, Queensland Institute of Medical Research, Brisbane 4029, Australia; 6 Griffith Medical Research College, a joint program of Griffith University and the Queensland Institute of Medical Research, Queensland 4029, Australia

## Abstract

**Background:**

HIV-1 reverse transcriptase (RT) is a heterodimer composed of p66 and p51 subunits and is responsible for reverse transcription of the viral RNA genome into DNA. RT can be post-translationally modified *in vitro *which may be an important mechanism for regulating RT activity. Here we report detection of different p66 and p51 RT isoforms by 2D gel electrophoresis in virions and infected cells.

**Results:**

Major isoforms of the p66 and p51 RT subunits were observed, with pI's of 8.44 and 8.31 respectively (p66_8.44 _and p51_8.31_). The same major isoforms were present in virions, virus-infected cell lysates and intracellular reverse transcription complexes (RTCs), and their presence in RTCs suggested that these are likely to be the forms that function in reverse transcription. Several minor RT isoforms were also observed. The observed pIs of the RT isoforms differed from the pI of theoretical unmodified RT (p66_8.53 _and p51_8.60_), suggesting that most of the RT protein in virions and cells is post-translationally modified. The modifications of p66_8.44 _and p51_8.31 _differed from each other indicating selective modification of the different RT subunits. The susceptibility of RT isoforms to phosphatase treatment suggested that some of these modifications were due to phosphorylation. Dephosphorylation, however, had no effect on *in vitro *RT activity associated with virions, infected cells or RTCs suggesting that the phospho-isoforms do not make a major contribution to RT activity in an *in vitro *assay.

**Conclusion:**

The same major isoform of p66 and p51 RT is found in virions, infected cells and RTC's and both of these subunits are post-translationally modified. This post-translational modification of RT may be important for the function of RT inside the cell.

## Background

The human immunodeficiency virus type 1 (HIV) reverse transcriptase (RT) enzyme catalyses reverse transcription of the viral RNA genome into double-stranded DNA in infected cells, a crucial early step in the virus life-cycle. RT is encoded by the Pol open reading frame, and is translated as a Gag-Pol protein precursor that is subsequently proteolysed by viral protease (PR) into 66 kDa (p66) and 51 kDa (p51) subunits with active RT formed as a heterodimer of p66 and p51 [1-3]. The p51 subunit shares the same N-terminal sequence but lacks the C-terminal 140 amino acids of p66. The subunits are functionally different: p66 possesses RNA-dependent and DNA-dependent DNA polymerase and RNase H activity, and p51 provides essential structural and conformational stability [4-7].

Reverse transcription of the viral RNA genome initially leads to synthesis of a 181 nt single-stranded, negative-sense DNA product called minus-strong stop DNA (-ssDNA) (reviewed in [8]). This first intermediate of reverse transcription is detected at low levels in a small proportion of intact virions [9-11] and although isolated intact HIV core structures can perform reverse transcription [12], following the entry of virions into cells, synthesis of -ssDNA and subsequent intermediate products of reverse transcription increases dramatically [13]. The -ssDNA subsequently hybridises to the 3' terminus of the viral genome (first strand transfer) allowing negative strand DNA synthesis to continue [14]. Plus strand DNA synthesis is initiated and following a second strand transfer, double-stranded viral DNA is completed. The kinetics of HIV reverse transcription during virus replication has been analysed in several studies [13-17], including a synchronous one-step cell-cell HIV infection model used in our laboratory which shows distinct time delays in the appearance of -ssDNA (1.5 hr post infection; pi), first strand transfer (2 hr pi) and second strand transfer DNA products (2.5 hr pi) [18]. The presence of these time delays during reverse transcription has suggested that recruitment or modification of cellular and viral factors and/or conformational changes in RT may be required for specific steps of the reverse transcription process [18].

Protein phosphorylation is known to regulate the enzymatic activity of a number of proteins including polymerases. Phosphorylation of RNA polymerase II (RNAPII) is essential for transition from the initiation to elongation phase of transcription [19], while de-phosphorylation of RNAPII is required for re-forming a competent RNAPII initiation complex [20]. Similarly, the HIV polymerase (or RT) may be regulated by phosphorylation. HIV RT can be phosphorylated *in vitro *by a number of kinases including auto-activated protein kinase (AK), myelin basic protein kinase (MBPK), cytosolic protamine kinase (CPK), casein kinase II (CKII) and protein kinase C (PKC) [21]. Furthermore, CKII-mediated phosphorylation of RT stimulates polymerase and RNase H activity *in vitro *[22] and recombinant HIV RT can be phosphorylated in insect cells [21]. Kinase-specific consensus sequences in HIV RT have also been found to be highly conserved within HIV subtypes [23,24]. Together, these results suggest that the RT process is activated during early infection, that RT is a substrate for phosphorylation and that phosphorylation may affect RT activity. We therefore investigated whether HIV RT underwent post-translational modification, specifically phosphorylation, during the progression of a normal HIV infection.

We report that RT p66 and p51 exist in virions and during HIV infection of cells as a number of protein isoforms, some of which are phosphorylated. The majority of RT is post-translationally modified and the major RT isoforms are present in HIV RTCs, suggesting that these isoforms play a biological function in the reverse transcription process inside the cell.

## Results

### Validation of pI measurements

We firstly verified that our 2D gel electrophoresis system could accurately measure small changes in pI by determining the theoretical and experimental pIs of recombinant histidine tagged (His)-RT and GAPDH. The theoretical pIs for unmodified recombinant His-p66, and His-p51 from the HIV LAI strain, RT_LAI _were calculated to be 8.53 and 8.60 respectively (Table 1). These calculated pIs were greater than 2 pH units above the pKa of His and thus the His-tag would reduce the pI of either protein by only 0.002 pH units, as estimated by ExPASy Compute, and produce a negligible shift in our 2D gel electrophoresis system. The theoretical pI's for RT_HXB2 _and recombinant RT_LAI _were the same (Table 1). The theoretical pI of GAPDH, used as an internal standard, was calculated to be 8.52. Additionally, we calculated the expected changes in pI for p66, p51 and GAPDH due to post-translational modification by phosphorylation or deamidation (Table 1). Other post-translational modifications such as acetylation could occur and would similarly induce an acidic shift in protein pI.

**Table 1:** Theoretical pIs of unmodified and modified RT containing phosphorylation or deamidations of 6His-tagged recombinant RT_LAI _(rRT) [37], RT_HXB2 _(Swiss-Prot: P04585), and GAPDH [42].

**Theoretical isoelectric point (pI)**						
**Protein**	**Unmodified**	**No. of Phosphorylation groups**			**Deamidations**	
		**1**	**2**	**3**	**1**	**2**
**rRT_LAI _p66**	8.53	8.16	7.60	7.19	8.36	8.13
**rRT_LAI _p51**	8.60	8.17	7.44	7.02	8.41	8.13
**RT_HXB2 _p66**	8.53	8.19	7.55	7.09	8.36	8.12
**RT_HXB2 _p51**	8.60	8.21	7.56	7.07	8.43	8.18
**GAPDH**	8.52	7.54	7.0	6.71	8.25	7.82

**Figure 1:**
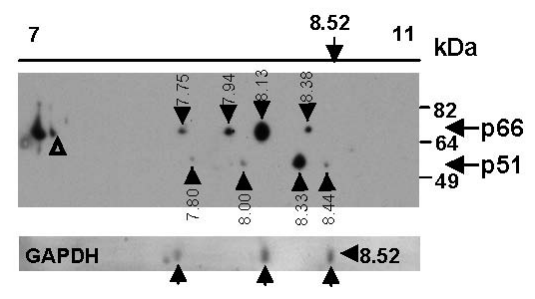
**2D gel electrophoresis analysis of recombinant RT identifies protein isoforms**. Recombinant RT_LAI _*+ *GAPDH protein (3 μg each) was solubilised in 2D gel electrophoresis buffer, focussed on a pH 7–11 non-linear, 11 cm Immobiline DryStrip gel then resolved on a 10% acrylamide SDS-PAGE gel followed by transfer to PVDF membranes. RT was detected by Western blot using an anti-RT antibody (upper panel) and GAPDH detected by Coomassie stain (lower panel). RT isoforms are designated by black arrows and calculated pI indicated. Position of *triangles *(Δ) denote the reference marks used for calculation of pI.

We determined the experimental pIs of purified recombinant RT_LAI _and GAPDH using 2D gel electrophoresis. RT was detected using western blot and GAPDH by Coomassie staining. A number of isoforms consistent in size with p66 or p51 were detected (Figure 1) with the major isoforms present having pIs of 8.13 and 8.33, respectively. The pIs of the most basic isoforms, p66_8.38 _and p51_8.44 _(Table 2), were lower than the theoretical pI values of unmodified p66_8.53 _and p51_8.6 _(Table 1), consistent with deamidation of a single asparagine residue calculated to change the pI by -0.17 and -0.19 pI units respectively (Table 1). The pI difference between p66_8.38 _and the major p66_8.13 _(-0.25 pI units) was consistent with a second deamidation predicted to affect the pI by -0.23 pI units (Tables 1 and 2). 2D gel electrophoresis analysis of GAPDH detected three isoforms by Coomassie staining (Figure 1). The major and most basic GAPDH isoform had an observed pI of 8.50 corresponding to the theoretical pI of unmodified GAPDH (8.52). The more negatively charged GAPDH isoforms had pI values -0.37 and -0.87 pI units lower than GAPDH_8.52_, consistent with singly and doubly deamidated forms of GAPDH with theoretical pI differences of -0.27 and -0.70 respectively (Table 1). These results are consistent with deamidation of both recombinant RT and GAPDH and demonstrate that changes in pI associated with post-translational modifications can be accurately measured using our 2D gel electrophoresis format.

**Table 2:** Observed pI of 6His-tagged recombinant RT_LAI _(rRT), and HIV-1 virion RT_HXB2 _p66 and p51 isoforms. Isoform in bold is the major isoform observed.

**Protein**	**Observed isoelectric point (pI)**			
**rRT p66**	8.38	**8.13**	7.94	7.75
**rRT p51**	8.44	**8.33**	8.00	7.80
**virion RT p66**	**8.44**	8.40	8.28	
**virion RT p51**	8.41	**8.31**	8.15	

### HIV RT exists as multiple isoforms

**Figure 2:**
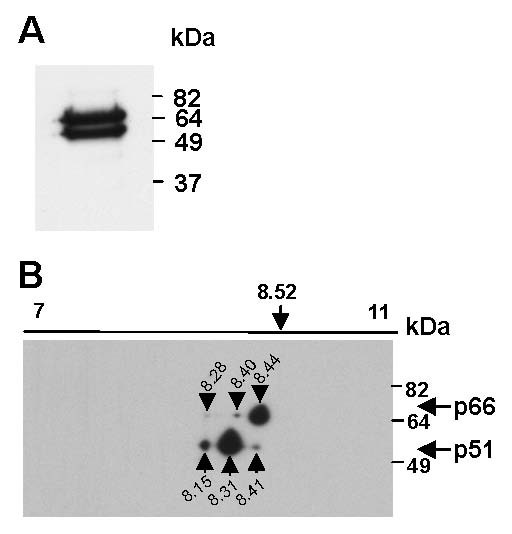
**RT isoforms are present in purified HIV virions**. Viral particles from H3B cells were pelleted through 25% sucrose, solubilised in 2D gel electrophoresis buffer and an aliquot resolved by 1D SDS-PAGE (A). The remaining sample was spiked with 3 μg of GAPDH protein, focussed on a pH 7–11 non-linear, 11 cm Immobiline DryStrip gel and then resolved by SDS-PAGE followed by transfer to PVDF membranes (B). RT was detected by Western blot using an anti-RT antibody. RT isoforms (B) are designated by black arrows and the calculated pI and expected position of p66 and p51 indicated.

To examine RT in purified HIV virus, HIV_HXB2 _virions were pelleted through 25% sucrose and then solubilised in 2D sample buffer. An aliquot was analysed by 1D SDS-PAGE and western blot for RT. As expected, two distinct bands corresponding to p66 and p51 were detected (Figure 2A). The remaining sample was then analysed by 2D gel electrophoresis. Three distinct isoforms of p66 and p51 were identified (Table 2). A summary of the reproducibly detected isoforms and potential post-translational modifications is presented in Table 3. The isoforms of virion p66_8.44 _and p51_8.31 _were most abundant and reproducibly seen (Figure 2B). Densitometric quantitation of images showed that these isoforms represented 85–90% of virion-associated RT (data not shown). The pIs of both of these major isoforms differed from that predicted for unmodified p66_8.53 _and p51_8.60_. The virion p51 isoforms showed a similar pI profile to the isoforms detected in recombinant RT, with the virion p51_8.31 _and p51_8.41 _isoforms similar to the recombinant p51_8.33 _and p51_8.44 _isoforms (Table 1). The minor RT isoforms suggest multiple modifications of p66 and p51 in HIV virions. The pI values for p51_8.41_and p51_8.15 _closely correspond to the theoretical pI's for RT_HXB2 _p51 deamidation (p51_8.43_, p51_8.18_, Table 1).

**Table 3:** Summary of the routinely observed isoforms of RT_HXB2_.

**Isoform**	**pI**	**Modification**
**p66**	**8.44**	**unknown**
	8.40	unknown
	8.28	phosphorylation + basic addition
	8.57	unmodified

**p51**	8.41	^a^phosphorylation + basic addition or ^b^deamidation
	**8.31**	^a^**phosphorylation + basic addition**
	8.15	^b^2 deamidations
	7.91	2 phosphates + basic addition

^a ^= de-phosphorylation observed in a one experiment only.

^b ^= based on theoretical pI (see table 1)

Major isoforms are highlighted in bold.

**Figure 3:**
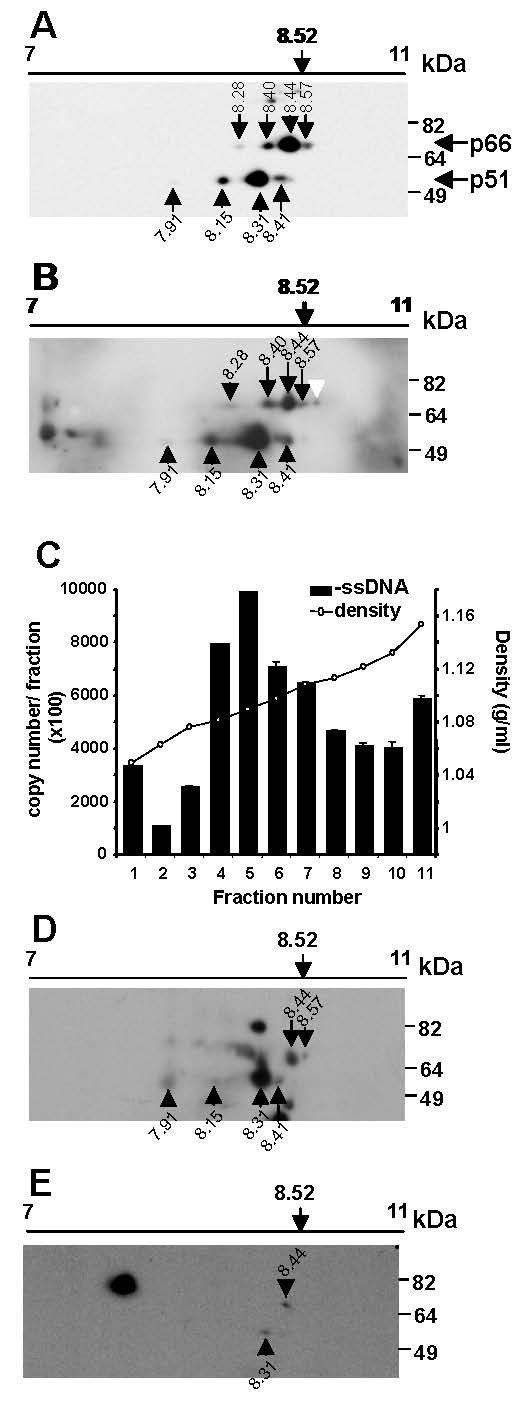
**The same major RT isoforms are present in virus producer cells, newly infected cells and HIV RTCs**. H3B and Hut-78 cells were co-cultured for the indicated time period then lysed. For panels A and B, lysates were immunoprecipitated using heat-inactivated AIDS patient sera cross-linked to protein A sepharose beads and washed. In panels A, B and D, E samples were subjected to 2D gel electrophoresis on a pH 7–11 non-linear, 11 cm Immobiline DryStrip gel along with 3 μg of GAPDH protein. Proteins were resolved by SDS-PAGE and transferred to PVDF membranes. RT was detected by Western blot using an anti-RT antibody and RT isoforms are designated by a black arrow (n = 2 for each panel). Minor differences in the p66 and p51 profiles were observed between experiments and spots not routinely observed are indicated by a white arrow. (A) H3B virus producer cells. H3B and Hut-78 cells were co-cultured and lysed immediately. (B) Infected cell lysates. H3B and Hut-78 cells were co-cultured and lysed at 40 min post-cell mixing. (C-E) HIV RTC's. Lysates were subjected to 15–30% sucrose velocity gradient sedimentation. Fractions (1 ml) were collected from the top of the gradient and viral -ssDNA analysed by real time PCR (C). The remainder of two selected fractions; (D) from the top of the gradient (fraction 1) and (E) co-incident with the known sedimentation of RTCs (fraction 5), were TCA precipitated and subjected to 2D gel electrophoresis, as for panels A and B, above. Experiments were replicated, at least n = 2, for each presented biological situation.

We next assessed the presence of these RT isoforms in other biological situations: in (i) virus producer cells (Figure 3A), (ii) intracellularly following HIV infection (Figure 3B) and (iii) in HIV RTC's (Figure 3C–E). H3B cells are chronically HIV infected cells that produce infectious virus and although they contain forms of HIV RT that are active *in vitro*, RT is not active inside the cell and newly synthesised HIV DNA is not formed until stimulation by mixing with uninfected recipient cells [2]. H3B cells thus represent a system to analyse changes in RT that occur co-incident with intracellular stimulation of reverse transcription and additionally offers the advantage of a synchronous and highly efficient infection model compared with a cell-free infection [13]. This allows high sensitivity in detecting RT protein. To analyse the RT in H3B producer cells we mixed H3B cells with uninfected Hut-78 cells and immediately lysed cells prior to the opportunity for interaction, stimulation of RT or infection. Proteins were then immunoprecipitated and subjected to 2D gel electrophoresis. p51_8.41_, p51_8.31_, p51_8.15 _and p51_7.91 _and p66_8.57_, p66_8.44_, p66_8.40_, p66_8.28 _isoforms were seen, representing RT present in H3B cells (Figure 3A). The two most abundant p66_8.44 _and p51_8.31 _isoforms had pI values identical to the two most abundant isoforms detected in virions (Figure 2B). Similar to that seen in virions, quantitation of western images indicated that these isoforms represented 76 ± 12 and 79 ± 2% of the p51 and p66 RT protein, respectively. New minor RT isoforms, not seen in virions were observed (p66_8.57 _and p51_7.91_) which for p66_8.57 _closely corresponds to the theoretical pI of unmodified p66_8.53_. Minor differences in the p66 and p51 profiles were observed between these and the subsequently described experiments which are likely attributable to variation in HIV infection, immunoprecipitation efficiency, and sensitivity of western blot detection and spots that were variably observed are indicated on the figures with a white arrow. A higher molecular weight RT immunoreactive species was sometimes observed (eg Figure 3A, 3D) which likely represents unprocessed Gag-Pol arising from the H3B producer cells.

We next analysed RT present after HIV infected H3B cells were mixed with uninfected Hut-78 cells at 37°C to allow virus entry and replication. The same two major p66_8.44 _and p51_8.31 _isoforms were again observed (Figure 3B). However, the relative proportions of the major and minor isoforms differed, with the minor isoforms becoming more prominent and the major p66_8.44 _and p51_8.31 _isoforms representing only 64 ± 11 and 60 ± 9% of the p51 and p66 RT protein, respectively. Similar minor isoforms were present in these cells undergoing active reverse transcription compared with those detected in chronically infected virus producer H3B cells.

After viral entry some RT remains part of a nucleoprotein complex termed the reverse transcription complex (RTC) but the majority of virion associated RT dissociates from the RTC [25]. We next assessed if specific isoforms of RT were associated with RTCs following HIV infection. Infections were initiated by cell-cell mixing as previously, and after 120 min, cell lysates were prepared and subjected to sucrose velocity gradient sedimentation. This sedimentation technique was chosen since we have previously observed that it yields good separation of free protein (fraction 1) and any remaining unactivated RT in pre-exisiting complexes from H3B cells (fraction 7) from RTCs (fraction 5) [2,26], the latter which we can monitor by virtue of the presence of newly synthesised reverse transcription products. HIV reverse transcription products showed a peak in gradient fraction 5 (1.08 g/ml sucrose; Figure 3C) consistent with the previously characterised sedimentation rate of RTCs as defined by the presence of newly synthesised DNA, RT activity and HIV integrase protein [26]. Sucrose gradient fractions were then subjected to 2D gel electrophoresis and western blot for RT, as above. Fraction 1 from the top of the gradient and containing free protein showed RT isoforms with migration characteristics consistent with p66_8.57_, p66_8.44 _and p51_8.41_, p51_8.31_, p51_8.15 _and p51_7.91_, with the major isoforms p66_8.44 _and p51_8.31 _(Figure 3D) as seen previously (Figure 2, 3A, 3B). However, in fraction 5 containing RTCs, only isoforms with migration characteristics consistent with p66_8.44 _and p51_8.31 _could be detected (Figure 3E). Although this does not exclude the presence of other less abundant RT isoforms in RTCs that were not detected due to the much lower levels of RT protein present, our results confirm that the major isoforms of p66_8.44 _and p51_8.31 _RT, seen in the virion and in infected cells, are associated with active RTCs and thus are the likely to be biologically relevant RT isoforms.

### Newly HIV infected cells contain phosphorylated isoforms of RT

**Figure 4:**
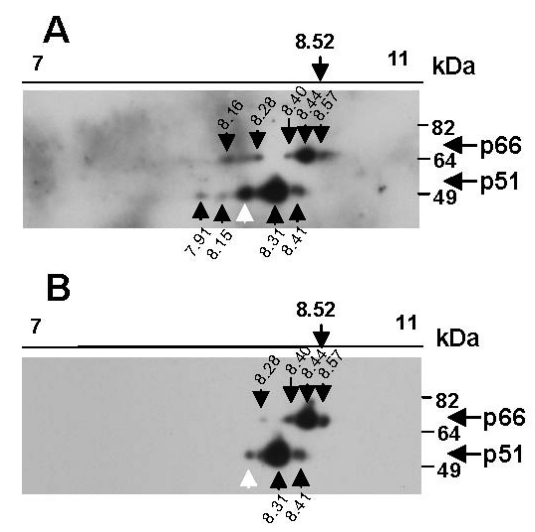
**Phosphatase treatment alters the RT isoforms detected**. H3B and Hut-78 cells were mixed and incubated at 37°C for 40 mins, cells were then lysed and virus protein immunoprecipitated using heat-inactivated AIDS patient antibody cross-linked to protein A sepharose beads. Immunoprecipitates were incubated without (A) or with (B) calf intestinal alkaline phosphatase (CIAP), proteins pelleted, washed and subjected to 2D gel electrophoresis on a pH 7–11 non-linear, 11 cm Immobiline DryStrip gel along with 3 μg of GAPDH protein, and then resolved by SDS-PAGE. RT was detected by Western blot using an anti-RT antibody. RT isoforms are designated by a black arrow and spots not routinely observed are indicated by a white arrow. Experiments were replicated (n = 3).

As one of the most important forms of protein modification is phosphorylation, we analysed the susceptibility of RT isoforms to phosphatase treatment prior to 2D gel electrophoresis. Validation of the efficiency of de-phosphorylation in our *in vitro *reactions was demonstrated by treating phosphorylated recombinant beta common (βc) chain of the GM-CSF receptor with phosphatase and confirming the loss of reactivity with anti-phospho-Ser-585βc polyclonal antibody by Western blot (data not shown) [27]. Next, HIV infection was initiated by mixing of H3B and Hut-78 cells and after 40 min the cells were lysed and viral proteins immunoprecipitated. Precipitated proteins were divided equally and treated with or without calf intestinal alkaline phosphatase (CIAP). The RT proteins were then analysed by 2D gel electrophoresis and detected by Western blot. The sample without phosphatase treatment showed a profile of p66 and p51 isoforms (Figure 4A) of calculated pI equivalent to p66_8.57_, p66_8.44_, p66_8.40_, p66_8.28_, and p51_8.41_, p51_8.31_, p51_8.15 _and p51_7.91 _as seen previously (Figure 2 and 3). Some additional minor p66 and p51 isoforms were also observed, again highlighting the experimental variation in the minor RT isoforms.

Removal of phosphate groups should increase protein pI if phosphorylation is present. Phosphatase treatment clearly altered the observed p66 and p51 isoforms (Figure 4B). The minor p66 isoforms, (p66_8.28 _and p66_8.16_) were greatly diminished or abolished by phosphatase treatment and this was reproducibly observed in replicate experiments, suggesting that these isoforms are phosphorylated. p66_8.16 _differed by -0.37 pI units from the theoretical pI of unmodified p66_8.53_, consistent with the -0.34 pI unit change associated with addition of a single phosphate group. This p66_8.16 _phosphorylated isoform was not routinely detected in all experiments. p66_8.28 _differed by -0.25 pI units from unmodified p66, suggesting that while p66_8.28 _is phosphorylated it also possesses additional modifications which make it more basic. p51_7.91 _was also consistently reduced by phosphatase treatment and differed by -0.69 pI units compared with unmodified p51, corresponding to a predicted addition of two phosphate groups and additional basic modification. Although most of p51 RT was relatively phosphatase resistant (Figure 4B) in one experiment phosphatase treatment reduced the levels of both p51_8.41 _and p51_8.31 _(data not shown). We have previously observed variation in de-phosphorylation and that total de-phosphorylation of ovalbumin is time-dependent; indicating slow removal of certain phosphate groups (CJ Bagley, unpublished results). Thus the variable susceptibly of some RT isoforms to de-phosphorylation may reflect reduced activity or restricted accessibility of the phosphatase enzyme to some phosphate groups present in the RT protein and thus we believe that p51_8.41 _and p51_8.31 _are most likely phosphorylated. Together the pI value and susceptibility to phosphatase treatment indicate that the RT isoforms p66_8.28_, p66_8.16 _and p51_7.91 _and potentially p51_8.41 _and p51_8.31 _are phospho-RT isoforms.

**Figure 5:**
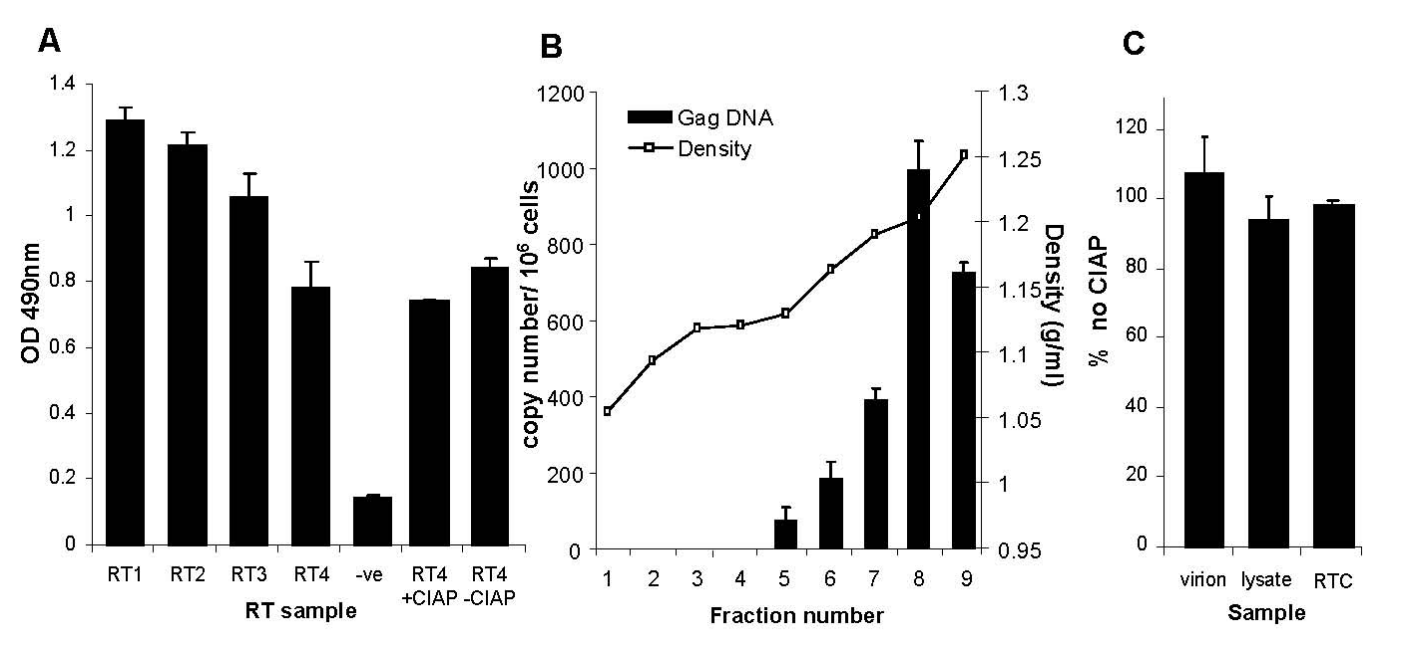
**Phosphatase treatment does not affect *in vitro *RT activity**. (A) Recombinant M-MuLV RT was assayed directly (RT1 = 500 milliUnits [mU], RT2 = 100 mU, RT3 = 20 mU, RT4 = 4 mU) or was incubated for 60 mins at 37°C in PBS or CIAP buffer +/- CIAP enzyme prior to exogenous RT activity assay, overnight at 37°C using DIG-UTP and colourimetric detection of incorporated DIG. (B) H3B and Hut-78 cells were co-cultured and lysed at 40 min post-cell mixing and lysates were subjected to 0–60% linear sucrose equilibrium gradient sedimentation. Fractions (1 ml) were collected from the top of the gradient and viral Gag DNA analysed by real time PCR. Fractions 7–8, containing HIV DNA and sedimenting at 1.19–1.25 g/ml sucrose was immunoprecipitated with AIDS patient sera and represented the RTCs used subsequently in the *in vitro *RT activity assay. (C) Samples from virions, cell lysates and RTCs were incubated for 60 mins at 37°C in PBS or CIAP buffer +/- CIAP enzyme prior to exogenous RT activity assay, overnight at 37°C using DIG-UTP and colourimetric detection of incorporated DIG. Results were normalized against the RT activity observed in the absence of CIAP and represents data from 3 independent dephosphorylation and RT activity assays and from 2 independent RTC preparations.

To analyse the significance of phosphorylated RT isoforms, cell lysates and virions were treated with or without phosphatase and RT activity was then assessed by *in vitro *exogenous RT activity assay (Figure 5). Since phosphatase itself could theoretically dephosphorylate dNTP's and influence the *in vitro *RT activity assay, we first validated measurement of RT activity in the presence of phosphatase and phosphatase buffering conditions. Incubation of recombinant M-MuLV RT in an *in vitro *RT activity assay in the presence of CIAP buffer alone or with CIAP enzyme had no effect on the quantitation of RT activity (Figure 5A). We next analysed the effect of phosphatase treatment on RT activity present in HIV virions, cell lysates and RTCs. RTCs were isolated by sucrose density gradient sedimentation, since this technique is best suited for concentrating particles into a more tightly sedimenting band than the velocity gradients used in Figure 3. Fractions 7–8, sedimenting at the previously defined density for RTCs [26] and containing newly synthesised reverse transcription products (Figure 5B,) were immunoprecipitated and subjected to dephosphorylation with CIAP, along with virions and cell lysates. Dephosphorylation reactions were performed as previously, which we know successfully dephosphorylates the βc chain of the GM-CSF receptor [27] and some isoforms of HIV RT (Figure 4). Dephosphorylation had no effect on the ability of RT found in virions, inside newly infected cells or associated with RTCs to perform *in vitro *reverse transcription (Figure 5C). Additionally, other sources of phosphatase; Antarctic phosphatase and lambda phosphatase similarly had no effect on RT activity of virions (data not shown), suggesting that phosphorylation makes limited contribution to the inherent activity of naturally occurring RT when measured in an *in vitro *assay.

## Discussion

Previous literature has suggested that RT may be subjected to post-translational modification, such as phosphorylation and it is well known that the process of reverse transcription is substantially activated upon cell infection. We thus hypothesised that this activation of RT may be related to its post-translational modification, particularly phosphorylation. In this study we have shown by 2D gel electrophoresis that modified RT forms are the major RT protein present in virions, newly infected cells and RTC's. The same predominant RT isoforms with pI's of p66_8.44 _and p51_8.31 _were seen in purified virions, intracellularly and associated with RTC's, and this suggests that these are the major biologically active RT form. The possibility that these represented an excess of inactive molecules present together with smaller levels of a modified active form, was considered unlikely since these forms predominated in semi-purified RTCs that are known to be supporting active reverse transcription. The major RT isoforms observed corresponded to an undefined post-translational modification for p66_8.44_, and potentially phosphorylation plus an undefined basic modification for p51_8.31_. The major p51_8.31 _isoform had lower pI than the major p66_8.44 _isoform, contrary to that seen for recombinant RT (p66_8.13 _and p51_8.33_) and the theoretical pI of the unmodified p51_8.60 _or p66_8.53_. Additionally, susceptibility of p51_8.31 _to phosphatase treatment in one experiment suggested that p51_8.31 _may be phosphorylated, while p66_8.44 _was phosphatase resistant in all instances. Thus the major p51_8.31 _isoform contains modifications that are different from those in the major p66_8.44 _isoform. This observed differential modification of p51 compared to p66 may be the result of (i) modification of a single p66 molecule of the RT homodimer that is then selectively targeted for cleavage giving rise to p51 and a mature RT heterodimer or (ii) selective modification of the p51 in the heterodimer post p66 cleavage. This differential modification of p51 and p66 may be important for selective regulation of RT enzymatic functions via p66 post-translational modifications or alterations to RT structure/conformation via post-translational modifications of p51. The identification of these RT isoforms is novel. Previous studies have identified at least two isoforms of MA and CA [28-30] in HIV virions by 2D gel electrophoresis analysis followed by silver stain or western blot, but these studies have not identified isoforms of RT, possibly due to lower levels of RT or the use of isoelectric focussing strips of insufficient resolving power for the pI range of RT [30,31]. The RT isoforms we observed changed little between virus producer cells, virions and newly infected cells, although the minor RT isoforms became more abundant following infection.

Some of the RT isoforms detected were phosphorylated, as suggested by their pI value and their susceptibility to dephosphorylation. Phosphorylation is known to modulate the activity of many proteins that interact with nucleic acids, including HIV proteins Tat, and Rev [32,33] and RNAPII [19,34]. Indeed phosphorylation of HIV RT *in vitro *led to increased polymerase and RNase H activities [21,22,35]. Similarly the phosphorylated forms of RT that we have identified may lead to p66/p51 heterodimers with different physical characteristics, activities or functionality and hence may play an important role in regulating reverse transcription in newly infected cells. Our results, however, show that dephosphorylation of RT from virions, cells lysates or RTCs had no effect on *in vitro *RT activity. This is not surprising given our results showing that the major isoforms that would be present in samples from virions, infected cells and RTCs are p66_8.44 _and p51_8.31 _that are not phosphorylated, and were phosphatase resistant in 2/3 experiments, respectively. Thus, naturally occurring phospho-RT isoforms are not a major contributor to RT activity, as measured *in vitro*, but could still be important for RT activity in the complex milieu of the infected cell, or may play a role in important structural interactions required for stability, movement and activity of the RTC intracellularly. Conclusive analysis of the roles of phosphorylation at specific sites in the RT enzyme remain to be determined by mutagenesis of potential RT phosphorylation sites and analysis of subsequent 2D gel electrophoresis profiles. However, at present this kind of analysis is hampered by the reduced sensitivity for detection of RT following infection with cell-free virus and 2D gel analysis, as would be necessitated in these experiments.

In conclusion, we describe for the first time the presence of modified p66 and p51 RT isoforms and report that the same major p51_8.31 _and p66_8.44 _isoforms are present in HIV virions, newly infected cells and active RTCs and thus are likely to be the forms playing a significant role in the reverse transcription process. The major p51_8.31 _and p66_8.44 _isoforms are modified differently, demonstrating selective modification of the RT subunits and although some RT isoforms are phosphorylated, phospho-isoforms of RT are not a major contributor to the inherent activity of RT, as measured in an *in vitro *activity assay. A better understanding of the post-translational modifications, the cellular enzymes involved and how these specifically influence RT activity inside the cell will be essential in elucidating the mechanisms for control of reverse transcription in newly infected cells.

## Methods

### Cells, virus and recombinant RT

H3B cells are a laboratory clone of H9 cells persistently infected with the HTLV-IIIB (HXB2) strain of HIV-1 [13]. Virus particles were isolated from clarified H3B cell culture medium by filtration (Sartorius, 0.22 μm filter), concentration (100,000 MwCO centrifugal filter, Millipore) and pelleting through 25% (w/v) sucrose at 86,500 g, 4°C for 1.5 hr (Beckman Optima™ TLX Ultracentrifuge). Recombinant RT (p6HRT; hexahistidine-tagged p66/p51 heterodimer, Dr. Nicolas Sluis-Cremer, University of Pittsburgh and derived from p6HRT-PROT [36]) was from the LAI sequence of HIV-1 [37] and produced by expression in M15 *Escherichia coli *and purified as described previously [38]. Purified recombinant RT was generously provided by Dr. Gilda Tachedjian, Burnet Institute, Melbourne, Australia.

### Cell-to-cell infection and lysis

H3B cells were mixed with Hut-78 cells at a ratio of 1:4 and incubated for 3 hr at 23°C to produce a temperature-arrested stage of infection [39]. Cells were then shifted to 37°C to allow infection to proceed. To extract protein, 1 × 10^8 ^cells were washed twice in ice-cold PBS and lysed by rotating at 4°C for 1 hr in 1 ml lysis buffer (5 mM Tris-HCl pH 7.4, 50 mM KCl, 0.05 mM spermine, 0.125 mM spermidine, 2 mM DTT, protease inhibitors [20 μg/ml aprotonin, complete mini protease inhibitor tablet (Roche), 2 mM PMSF), phosphatase inhibitors (2 mM NaF, 10 mM sodium pyrophosphate, 2 mM sodium orthovanadate], and 0.2% (v/v) Triton X-100). The cell lysate was clarified twice by centrifugation at 17,000 g/4°C for 30 min before immunoprecipitation.

### Immunoprecipitation of viral protein from infected cell lysate

Sera from four HIV-1 positive patients were pooled and heat-inactivated (AIDS patient sera (APS)) and incubated with protein A sepharose CL-4B beads (Pharmacia) at 4°C, rotating for 16 hr. Antibody was cross-linked to protein A using 5 mg/ml dimethyl pimelimidate (DMP) (Pierce) as described previously [40]. To immunoprecipitate viral proteins, cell lysates were incubated with APS-protein A sepharose CL-4B for 16 hr rotating at 4°C. The beads were then pelleted by low-speed centrifugation and washed in ice-cold water three times then proteins eluted directly into 2D gel electrophoresis buffer (see below).

### Fractionation of HIV reverse transcription complexes

HIV RTCs were fractionated on sucrose gradients as described previously [26,41]. Briefly, infections were initiated by mixing of H3B and Hut-78 cells, as described above. At 120 min post mixing cells were harvested, washed, lysed in buffer containing 0.1% (v/v) Triton X-100 and subjected to 15–30% sucrose velocity gradient sedimentation or 0–60% sucrose equilibrium density gradient sedimentation. 1 ml fractions were collected from the top of the gradient and 1/10^th ^of each fraction was analysed for HIV reverse transcription products by real time PCR. The remainder of the velocity gradient fractions were TCA precipitated and 85 μg of the total protein from each fraction was subjected to 2D gel electrophoresis, as below.

### 2D gel electrophoresis and Western blot analysis of protein

Samples were solubilised directly in 2D buffer (7 M urea, 2 M thiourea, 2% (w/v) CHAPS, and 0.5% pH 7–11 NL carrier ampholytes) and spiked with 3 μg glyceraldehyde-3-phosphate dehydrogenase (GAPDH, from rabbit muscle, Sigma) and 65 mM DTT. Samples (100 μl) were loaded, by anodic cup loading, onto a pH 7–11 non-linear, 11 cm Immobiline DryStrip (GE Healthcare) gel which had been hydrated in 2D sample buffer containing 1.2% (v/v) 2-hydroxethyldisulfide. Gels were run in a step-wise voltage gradient: 0–300 V/2 hr; 300–500 V/2 hr; 500–1000 V/2 hr; 1000–4000 V/5 hr followed by 4000 V/3 hr and then maintained at 500 V. Total volt hours (V/hr) ranged between 25–30,000 V/h. Focused proteins from individual gel strips were then separated by SDS-PAGE, using a 10% or 12% gel with a 29:1 acrylamide:bis-acrylamide ratio, alongside BenchMark™ prestained protein markers (Invitrogen), before transferring to PVDF transfer membrane (Hybond™-P; GE Healthcare). Membranes were blocked for 1 hr in TBST (50 mM Tris pH 7.4, 135 mM NaCl, 0.1% (v/v) Tween-20) containing 5% (w/v) skim-milk powder before incubating with rabbit anti-RT antibody (1:5000 dilution), (NIH AIDS Research and Reference Reagent Program, Dr. Stuart Le Grice, Division of AIDS, NIAID, NIH). Bound antibody was detected using horseradish-peroxidase-conjugated goat anti-rabbit IgG secondary antibody, and visualised using Super Signal West Dura Extended Duration Substrate (Pierce) and Kodak BioMax film (Integrated Sciences). To determine the relative proportion of p66 and p51 isoforms, protein spots in were quantitated by volume integration (Imagequant v3.3, Molecular Dynamics) and expressed as a percent of the total intensity of signal for RT p66 or p51.

### Phosphatase treatment of viral proteins

Viral proteins were immunoprecipitated from infected cell lysates with APS conjugated protein A sepharose CL-4B beads as described above, virions were prepared by PEG precipitation of high titre virus supernatant, and RTCs were prepared by equilibrium gradient sedimentation, as above. One half of each sample was treated with 40 units of calf intestinal alkaline phosphatase (CIAP; Promega) in CIAP buffer; (50 mM Tris, pH 9.3, 1 mM MgCl_2 _0.1 mM ZnCl_2 _and 1 mM spermidine and protease inhibitors (20 ug/ml aprotonin, complete mini protease inhibitor tablet [Roche], 2 mM PMSF). The other half was resuspended in CIAP buffer, protease and phosphatase inhibitors (2 mM PMSF, 2 mM NaF, 10 mM sodium pyrophosphate, 2 mM sodium orthovanadate). Reactions were incubated 37°C for 1.5 hr. For subsequent 2D gel analysis, bead bound samples from cell lysates were pelleted, washed in ice-cold water three times and the bound virus protein was eluted in 2D gel electrophoresis sample buffer. For subsequent RT activity assay, reactions were used directly, without further processing.

### RT activity assay

RT activity was quantitated *in vitro *using an exogenous activity assay. Briefly, microtitre plates (Covalink, Nunc) were coated with poly-A (Roche) then incubated with RT mix containing the test sample with 4.2 μM Digoxigenin (DIG)-UTP (Roche Diagnostics) and 2.5 μg/ml Oligo dT_12–18 _(GE Healthcare) in 8.4 μM dTTP, 25 mM KCl, 6.25 mM MgCl_2_, 62.5 mM Tris, pH 7.8, 1.25 mM DTT, 0.1% (v/v) Triton X-100, overnight at 37°C. Polymerised DIG-UTP was detected with anti-DIG-HRP conjugate (Roche Diagnostics, at 1/2500 dilution), reacted with 3,3',5,5'-tetramethylbenzidine (TMB substrate, Sigma) and quantitated by measurement of OD at 490 nm. Recombinant Moloney Murine leukemia virus (M-MuLV, New England Biolabs) was used as a comparative standard.

### Estimation of protein isoelectric point

The distance migrated along the IEF strip from the loading point (anodic, pH 7 end) was measured as a percentage of the total gel-strip length (11 cm) and the pI calculated from an idealised pH 7–11 non-linear migration reference graph (GE Healthcare). For internal calibration, GAPDH was spiked into individual viral protein samples before focusing and small puncture holes made in the PVDF membrane were used to align the Coomassie-stained and the Western blot images. Theoretical pI values for unmodified HIV_HXB2 _p66 and p51 (Swiss-Prot: P04585), recombinant hexahistidine-tagged p66 and p51 proteins, and GAPDH [42], with one or more phosphate or deamidation modifications, in 8 M urea, were calculated using pKa values as used by the ExPASy Compute pI/Mw tool  with the assumption that the pKa values of the protein's phosphate groups were 2.1 and 7.2.

## Competing interests

The authors declare that they have no competing interests.

## Authors' contributions

JMC performed isolation and analysis of RTCs, the dephosphorylation and RT activity experiments, contributed to interpretation of results and was the primary manuscript author, AJD was the main research worker and performed the 2D gel analysis experiments and pI calculations, CJBagley assisted in interpretation of all 2D gel electrophoresis and pI calculations, JP contributed in the design of CIAP experiments, DW, DH, CJBurrell and PL contributed to the design of the study. All authors read and approved the final manuscript.
